# Evidence from Multiple Species that Spider Silk Glue Component ASG2 is a Spidroin

**DOI:** 10.1038/srep21589

**Published:** 2016-02-15

**Authors:** Matthew A. Collin, Thomas H. Clarke, Nadia A. Ayoub, Cheryl Y. Hayashi

**Affiliations:** 1 University of California, Riverside, Department of Biology, Riverside, California 92521, United States; 2 Washington and Lee University, Department of Biology, Lexington, Virginia 24450, United States

## Abstract

Spiders in the superfamily Araneoidea produce viscous glue from aggregate silk glands. Aggregate glue coats prey-capture threads and hampers the escape of prey from webs, thereby increasing the foraging success of spiders. cDNAs for Aggregate Spider Glue 1 (ASG1) and 2 (ASG2) have been previously described from the golden orb-weaver, *Nephila clavipes*, and Western black widow, *Latrodectus hesperus*. To further investigate aggregate glues, we assembled *ASG1* and *ASG2* from genomic target capture libraries constructed from three species of cob-web weavers and three species of orb-web weavers, all araneoids. We show that ASG1 is unlikely to be a glue, but rather is part of a widespread arthropod gene family, the peritrophic matrix proteins. For ASG2, we demonstrate its remarkable architectural and sequence similarities to spider silk fibroins, indicating that ASG2 is a member of the spidroin gene family. Thus, spidroins have diversified into glues in addition to task-specific, high performance fibers.

Spiders have evolved numerous types of webs for prey capture that vary in geometry, mechanism, and constituent proteins. One of the most well-studied capture webs is the wagon-wheel shaped orb-web spun by spiders in the superfamily Araneoidea. Dominant architectural elements in these webs are the frame, radii, and the sticky capture spiral. Spider silks are synthesized in abdominal silk glands, and the silk glands of araneoid spiders are variously specialized to produce a particular type of silk. For example, major ampullate silk glands are the source of major ampullate fibers that are used in the frame and radii. Similarly, flagelliform silk glands produce the filament of the capture spiral while aggregate silk glands secrete sticky aggregate glue.

Each silk gland is connected to its own extruding spigot on the exterior of the spider’s spinnerets. The spigots for flagelliform and aggregate silk glands are adjacent to each other on the posterior lateral spinnerets, and a spider secretes both silks simultaneously when spinning the capture spiral^1^. Aggregate silk beads into evenly spaced droplets along the length of the flagelliform silk fibers^2^. When an insect impacts a web, sticky droplets behave as viscoelastic solids that not only adhere to the prey, but also elongate to maintain a physical connection between the struggling prey and the web^3^,^4^. For example, a single viscous droplet from the orb-weaving spider, *Larinioides cornutus*, can withstand up to 400 μN of pull off force and extend up to 800 μM^3^. However, the adhesive capability of spider webs is even greater as the close spacing of the droplets along the length of the capture spiral filament ensures that multiple droplets contact the prey. Each additional contacting viscous droplet increases the force necessary for an insect to pull free from the web^5^.

Compound light microscopy images of the capture spiral reveal three “layers” of viscid silk within a viscous droplet: a core, an aqueous layer, and an outer layer. The inner core is a fibrous nodule of aggregate gland secretions that encase flagelliform silk fibers^3^,^6^. It is thought that the nodule transfers the force of the captured prey to the axial fibers^3^. The aqueous middle layer is a sticky globe of glycoproteins, low molecular weight ionic compounds, and neurotransmitters (e.g. choline, isethionic acid, GABamide), collectively called “salts,” that coats the nodule^2^. The hygroscopic salts absorb ambient humidity, which allows the glycoproteins to remain mobile, thereby enhancing the adhesive properties of droplets^2^,^7^. Additionally, the collected water keeps the flagelliform fibers pliable^8^. The outer layer is characterized as being more fluid than the inner glycoprotein layer and contains fatty acids that may attract prey^3^.

Aggregate silk gland secretions have been greatly understudied at the genetic level. In fact, aggregate silk gland complementary (c) DNAs and proteins have been characterized from only two species, the golden orb-weaver *Nephila clavipes*^9^ and the Western black widow *Latrodectus hesperus*, which builds three-dimensional cob-webs^10^,^11^. The first reported aggregate gland cDNAs from *N. clavipes* were considered to be full-length and proposed to encode sticky components of the viscous glue droplets, aggregate spider glue 1 (ASG1) and 2 (ASG2)^9^. Both the ASG1 and ASG2 amino acid sequences are predicted to have several glycosylation sites, consistent with the observation that *N. clavipes* viscous glue droplets contain glycosylated proteins^9^.

An unusual feature of the *N. clavipes ASG1* and *ASG2* transcripts is that they contain a 353 base pair (bp) repetitive region that is present in *ASG1* in one orientation and present in *ASG2* in the reverse complement orientation^9^. The repetitive region of *ASG1* encodes iterations of an amino acid sequence motif that is dominated by glutamic acid, threonine and proline. This protein region is analogous to vertebrate mucin domains, which are glycosylated^9^,^12^. In *ASG2*, the reverse complement of the 353 bp repetitive region translates into iterated motifs that are rich in serine, glycine and valine. The related sequences in ASG1 and ASG2 were interpreted as evidence for, “two proteins expressed from opposite strands of the same DNA sequence”^9^. This suggests that the 353 bp region could be derived from a common exon that is transcribed in opposite directions (i.e., overlapping genes)^13^.

Later, a study of *L. hesperus* aggregate glands described cDNAs for aggregate silk factors 1 (AgSF1) and 2 (AgSF2), which function to cement web fibers and wrap prey^10^. Unlike ASG1 and ASG2, AgSF1 and AgSF2 are not thought to form glue droplets. Subsequently, a transcriptome study of *L. hesperus* silk glands identified homologs to the *N. clavipes ASG1* and *ASG2*^11^. The *L. hesperus ASG1* transcript has sequence that corresponds to the *N. clavipes* 353 bp region, but the *L. hesperus ASG2* transcript is devoid of any matching sequence to that region, in any orientation. The lack of shared sequence between *L. hesperus ASG1* and *ASG2* transcripts, as described for the *N. clavipes ASG1* and *ASG2* cDNAs, may be due to alternative splicing, misassembled transcripts for either *N. clavipes* or *L. hesperus*, or a taxon specific insertion/deletion event.

To assess the aggregate glue genes of cob-web and orb-web weaving spiders, we characterized *ASG1* and *ASG2* from the genomes of multiple species. Furthermore, gene sequences can reveal exon-intron structures, particularly in relation to the 353 bp region that is present in opposite orientations in the published *N. clavipes ASG1* and *ASG2* cDNAs^9^, but absent from *L. hesperus ASG2*^11^. To identify homologs of *ASG1* and *ASG2,* we constructed genomic target capture next generation libraries. We then conducted comparative analyses to identify key features of spider silk glue.

## Results and Discussion

### *ASG1* has Introns

**Figure 1:**
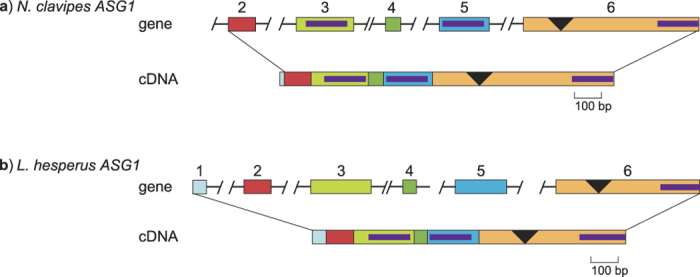
Comparison of *ASG1* gene models to cDNAs for *N. clavipes* (**a**) and *L. hesperus* (**b**). Boxes represent exons and horizontal lines represent introns. Exons are numbered. Lines show the correspondence of exons to cDNA regions. Slashes indicate unknown sequence between exons. Inverted triangles represent the mucin-like domain, and purple bars represent chitin binding domain 2 (see text). Exon 1 of *N. clavipes ASG1* was not captured, but is predicted from the cDNA. GenBank accession numbers are in Table 1.

Multiple (4–11) contigs that match regions of published *ASG1* were identified from each target capture species (Table 1). For *N. clavipes*, none of the contigs contains an entire *ASG1* coding region. Instead, the multiple contigs represent exons and portions of flanking introns (Fig. 1a). These contigs are predicted to have exon acceptor and/or donor splice junctions with probabilities greater than 60%. Genomic contigs of exons two through six match the cDNA with 94% sequence identity along the 1,184 bp length of concatenated exons, indicating that the cDNA and genomic contigs are correctly assembled. Furthermore, given the correspondence to our genomic contigs, the cDNA available for *N. clavipes ASG1* contains only 15 bp of what we now know is exon 1 and no 5′ UTR.

The longest *N. clavipes ASG1* genomic contig corresponds to the sixth exon, which is the final and longest exon, coding for about half of the mRNA transcript. This exon includes a chitin binding domain and the 353 bp repetitive region that was reported to be shared by *ASG2*, but in the reverse complement orientation^9^. However, our genomic contigs show that the 353 bp region is not isolated in a separate exon. Furthermore, if reverse complemented, the coding region of the final exon of *ASG1* extends beyond the ASG2 repetitive region and codes for an additional 34 amino acids that is not present in the ASG2 cDNA. Thus *N. clavipes ASG1* and *ASG2* cannot be overlapping genes.

For *L. hesperus*, contigs representing all six exons were recovered. When concatenated, these exons support the full-length cDNA transcript (Fig. 1b; 92% sequence identity over 1,152 bp). The final exon in *L. hesperus* is 603 bp, representing about 50% of the mRNA transcript length. This exon is shorter than the corresponding *N. clavipes* exon because it has fewer iterated repeats in the repetitive region.

### *ASG1* is Multi-locus

For the other target capture species, only contigs corresponding to *ASG1* exon 6 could be definitively identified. All contigs with a complete sixth exon began with the 3′ portion of the preceding intron, followed by a predicted exon acceptor splice junction, coding region, and 3′ UTR sequence. Since the sixth exon is several hundred bases long, numerous target capture probes covered this region, consistent with the recovery of this exon from all species.

**Figure 2:**
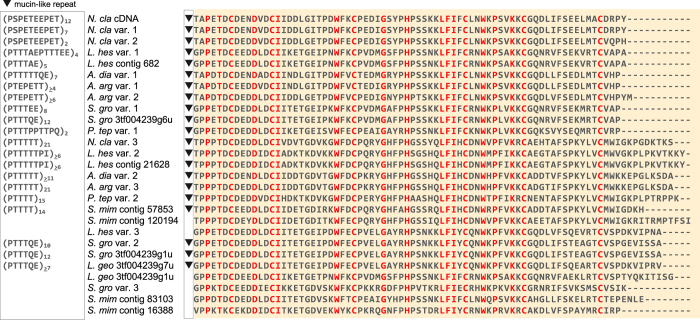
Alignment of the carboxyl-terminal region of ASG1 homologs. Amino acids are depicted by single letter IUPAC abbreviations. Red indicates amino acids conserved in all sequences. Black inverted triangles represent the presence and location of mucin-like domains; sequences lacking a triangle do not have mucin-like domains. Exemplar mucin-like repeats with subscripts denoting the number of tandem repeats are in the box to the left of sequence names. “≥” indicates that only a partial-length repetitive region is known. Species names are abbreviated. Full species names and GenBank accession numbers are in Table 1.

Two or three *ASG1* exon 6 homologs were recovered per target capture species and additional homologs were identified from NCBI databases (translations shown in Fig. 2). Considering the number and sequence divergence of exon 6 variants within a species, this means that *ASG1* is a member of a diversified gene family. For example, *N. clavipes* has three known exon 6 variants, and they share 52–95% pairwise nucleotide sequence identity within an individual spider genome. Because spiders are diploid, there must be at least two *ASG1* loci underlying these variants.

**Figure 3:**
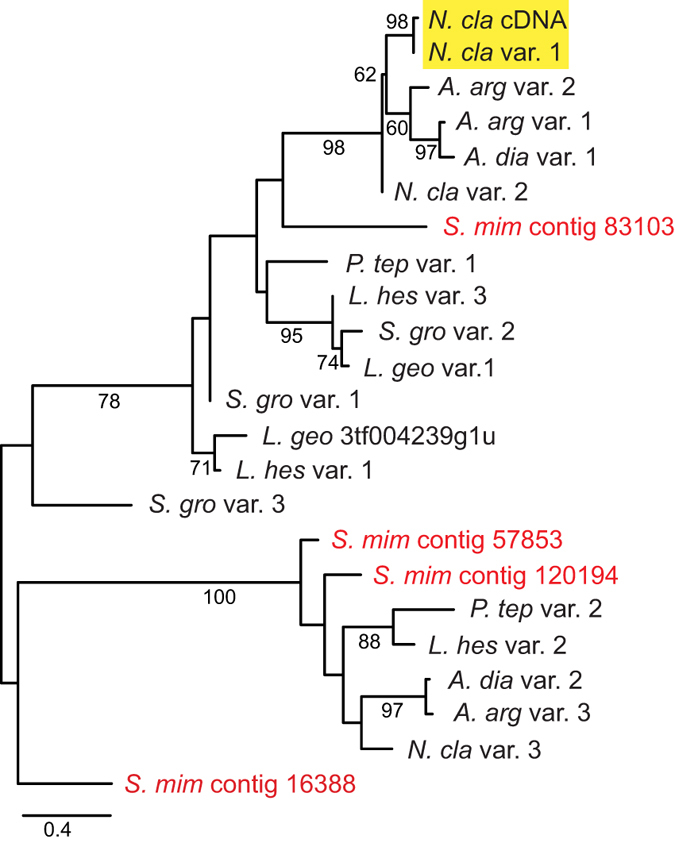
Maximum likelihood tree of the carboxyl-terminal region of ASG1 homologs. Highlighted box shows *N. clavipes* ASG1 cDNA and the most closely related ASG1 target capture contig. When there was an identical match between a translated genomic contig and a cDNA transcript, only the genomic contig is shown in the tree (*L. hes* var. 1 = *L. hes* contig 682, *L. hes* var. 2 = *L. hes* contig 21628, *S. gro* var. 1 = *S. gro* 3tf004239g6u, *S. gro* var. 2 = *S. gro* 3tf004239g1u). Red names indicate contigs from the *Stegodyphus mimosarum* genome. Full species names and GenBank accession numbers are in Table 1. Bootstrap percentages >50% are shown. Scale bar indicates 0.4 substitutions per site. Tree is mid-point rooted.

A phylogenetic analysis of the conserved carboxyl-terminal region alignment (Fig. 2) reveals the relationships of ASG1 variants within a genome to each other and to previously published sequences (Fig. 3). Focusing on the *N. clavipes* sequences, the ASG1 variants belong to two separate clades. One clade includes the pairing of the published *N. clavipes* cDNA^9^ and our *N. clavipes* variant 1 (Figs. 1a and 3 yellow box), as well as *N. clavipes* variant 2. The translated cDNA and genomic variant 1 differ by only a single amino acid replacement and we interpret them as alleles at the same locus. Genomic variants 1 and 2 are 93% identical at the amino acid level (79 out of 85 amino acids, Fig. 2) and have 84% nucleotide identity in the 3′ UTR. Given these similarities, variants 1 and 2 may also be alleles at the same locus. *N. clavipes* variant 3, however, is distantly related to *N. clavipes* variants 1 and 2, sharing a scant 47% average amino acid identity with them (Fig. 2) and 27.9% average nucleotide identity in 3′ UTR. Thus, variant 3 is attributed to a second locus.

### *ASG1* is Not Specific to Aggregate Silk Glands

Homologs of ASG1 were found in the *Stegodyphus mimosarum* genome (Fig. 3, red). *S. mimosarum*, a social velvet spider, is in the family Eresidae, which is distantly related to Araneoidea. Aggregate silk glands are restricted to Araneoidea, thus *S. mimosarum* spiders have neither aggregate silk glands nor are known to produce viscous glue. Instead *S. mimosarum* spiders use silk nanofibrils that are Velcro-like for prey capture^14^. Given their lack of aggregate silk glands and lack of viscous glue use, *S. mimosarum* is not expected to have genes for aggregate silk glues. Nevertheless, ASG1 homologs were identified from the *S. mimosarum* genome and are interspersed with the araneoid ASG1 homologs (Fig. 3). The presence of ASG1 homologs in *S. mimosarum* suggests that ASG1 does not have a function specific to the production of aggregate glue.

Several of our theridiid *ASG1* target capture contigs match published cDNA transcripts from *L. hesperus*, *S. grossa,* and *L. geometricus* (Fig. 2)^11^,^15^. Each of these cDNAs unequivocally clusters with their corresponding target capture contig (Fig. 3). In an analysis of tissue-specific RNA-seq libraries, transcriptional activity was observed for *ASG1* variants in both *L. hesperus* silk glands and non-silk gland tissues^11^. In fact, *ASG1* expression level was higher in cephalothorax than in silk glands. For example, the expression level of *L. hesperus ASG1* variant 1 (GBCS01010755) was three-fold higher in cephalothorax than in silk glands. Thus, *ASG1* is not exclusively expressed in silk glands, further evidence that this gene does not encode a viscous glue component.

### Potential Function of ASG1 as a Peritrophic Matrix Protein

To infer the function of ASG1, we examined the alignment of the translated exon 6 from the various homologs (Fig. 2). Invariant amino acids were found across all homologs (highlighted in red, Fig. 2). These amino acids correspond to a conserved chitin binding domain type 2 (ChtBD2)^9^,^12^. As part of ChtBD2, the six cysteine (C) residues in each sequence are predicted to form three sets of disulfide bonds that link β-sheets within the domain into a chitin binding pocket. Similarly, the conserved aromatic residues (phenylalanine (F) and tryptophan (W) depicted in red, Fig. 2) are predicted to be important for binding chitin^12^.

Immediately upstream of ChtBD2, 21 of the 27 ASG1 homologs have a complete or partial-length region of iterated repeat units dominated by proline (P) and threonine (T) residues, referred to as the mucin-like domain (triangles, Figs. 1 and 2). The mucin-like repeats include other polar amino acids, such as serine, glutamine, or glutamic acid (S, Q, E, respectively, Fig. 2 left panel). The β-turns resulting from the proline residues in mucin-like domains could expose the glycosylated threonine and serine residues by extending them outward, away from bound chitin.

We further investigated the potential function of ASG1 with DELTA-BLAST^16^. DELTA-BLAST search of the nr database with the translated *N. clavipes* ASG1 cDNA (EU780014) corroborated the previous identification of three chitin binding domains^9^. The top hit after the query itself was an insect peritrophic matrix intestinal mucin from the diamondback moth *Plutella xylostella* (AAN63949; 84% coverage, e-value 7e-49). Peritrophic matrix intestinal mucin proteins bind to chitin to coat anatomical structures, such as the walls and linings of midgut epithelium and glands in insects^17^. Thirty-one copies of peritrophic matrix intestinal mucin genes have been identified within the *Tribolium castaneum* (red flour beetle) genome, and multiple copies have also been found in other insect genomes^18^. Thus, peritrophic matrix genes comprise a multi-gene family and homologs have been identified within several arthropod lineages outside of insects, including Chelicerata^18^,^19^.

Insect intestinal mucins, a subtype of peritrophic matrix protein, characterized from *Mamestra configurata* (Bertha armyworm) contain two to five Cht2BDs and some have mucin domains high in threonine (up to 68.7%), serine (13.5%) and proline (18.1%)^17^. ASG1 also has Cht2BDs and mucin-like domains that are high in threonine, serine, and proline^12^. Indeed, CDART^20^, which searches the NCBI database based on placement and number of conserved protein domains, identifies the entire translated products of ASG1 cDNAs from both *L. hesperus* (GBCS01010755) and *N. clavipes* (EU780014) as mucin-like proteins.

ASG1 has ChtBD2 domains, implying that there is an interaction with chitin. Chitin is a common component of arthropod bodies. In fact, chitin has been detected within the spider *Nephila edulis* where it has been implicated in stiffening the distal portion of the major ampullate silk gland duct to create shear forces for fiber formation and to reinforce the duct walls as internal pressure increases^21^. Histochemical staining and Fourier transformed infrared spectroscopy of *N. edulis* silk gland ducts and the hindgut revealed the same chitin signatures^21^. Furthermore, chitin was observed in the extracellular matrix within the distal portion of *L. hesperus* major ampullate silk gland^22^. Chitin use in silk glands is not limited to spiders, chitin is present in the convergently evolved silk gland ducts of the domesticated silkworm *Bombyx mori*^21^. Thus ASG1 likely binds chitin in the silk gland wall and ducts, explaining the presence of *ASG1* transcripts in *N. clavipes* aggregate silk gland mRNA^9^. However, given the higher expression of ASG1 in non-silk tissues than silk-glands of *L. hesperus*^11^, we posit that ASG1 is not a silk glue but rather is a homolog of an insect intestinal mucin that functions in multiple locations throughout a spider.

### *N. clavipes* ASG1 and ASG2 are unrelated

Target capture was also successful for *ASG2*. For *N. clavipes*, there was one *ASG2* contig per library, with no nucleotide variation between libraries. We report the longer contig (2,821 bp vs. 2,768 bp), which contains 2,391 bp of continuous coding sequence, followed by a stop codon and then 430 bp of 3′ UTR (KU132353). Compared to the *ASG2* cDNA (EU780015), this genomic contig extends 246 bp farther upstream, with no evidence of a splice junction, start codon, or 5′ UTR. Hence, the 5′ coding region of *N. clavipes ASG2* remains unknown, and the *ASG2* cDNA is not a full-length transcript, as was originally proposed^9^.

**Figure 4:**
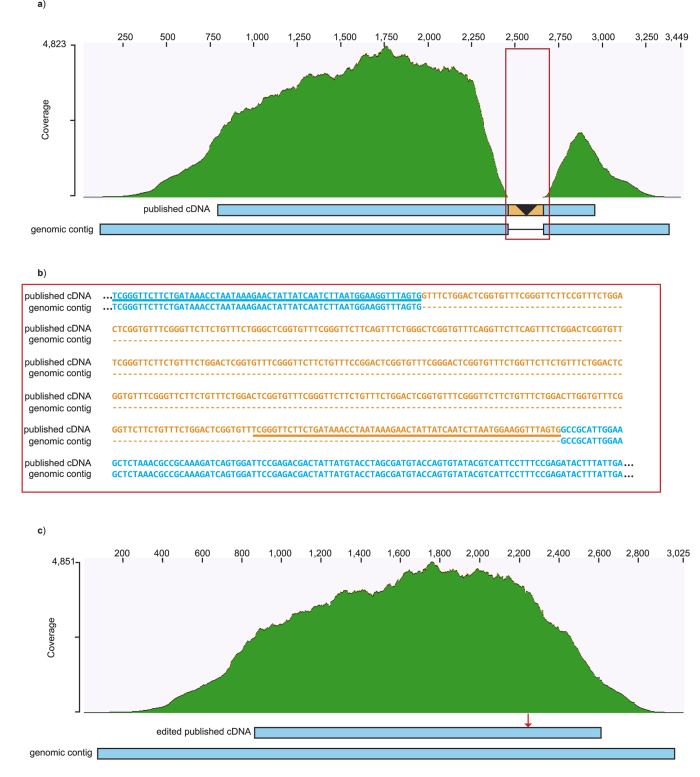
Number of paired-end reads (coverage) from an *N. clavipes* target capture library mapped to *N. clavipes ASG2* sequences. (**a**) Read coverage mapped to the published cDNA and corresponding capture contig of genomic DNA. Long dash is an alignment gap. Note the absence of reads to the 423 bp insertion (red boxed region, see text). (**b**) Alignment of published cDNA to genomic contig within the boxed region shown in **a**. Matching sequence in blue, insertion sequence in orange. Underlined segments are directly repeating sequence in the cDNA. (**c**) Read coverage mapped to the edited cDNA and capture contig. Red arrow indicates where sequence was removed from the cDNA. Y-axes are coverage depth in number of sequences and X-axes is nucleotide position from 5′ to 3′. GenBank accession numbers in Table 2.

Further comparison of the *N. clavipes ASG2* genomic contig to the published cDNA reveals a major discrepancy. The genomic contig lacks the 353 bp region purportedly shared with *ASG1*^9^. Since the genomic *ASG2* sequence lacks splice junctions, the aberrant region in the published cDNA cannot be attributed to alternative splicing. Instead, alignment of the *ASG2* genomic contig to the *ASG2* cDNA requires the insertion of a 423 bp gap in the genomic sequence (Fig. 4a red box, Fig. 4b). The corresponding region in the cDNA contains the 353 bp reverse complemented sequence from *ASG1* plus an additional 70 bp of downstream sequence (Fig. 4a orange rectangle, Fig. 4b orange text). Furthermore, flanking each side of this region in the cDNA is a copy of a 60 bp direct repeat (Fig. 4b underlined blue and orange bases). These direct repeats in the cDNA share 100% nucleotide identity to each other. By contrast, the genomic contig has only one copy of this 60 bp unit.

To investigate whether any capture library sequencing reads support the discrepant 423 bp (Fig. 4b), the *N. clavipes* paired-end reads were mapped to the *ASG2* cDNA. Dramatically, there is a complete absence of reads mapping to the 423 bp region (Fig. 4a red box). We pruned this region from the cDNA, redid the read mapping, and found that the reads mapped well over the entire lengths of the edited cDNA and genomic contig (Fig. 4c). Thus, the assembled genomic contigs and mapped genomic library reads do not support the existence of the serine/glycine rich encoding region present in the published *ASG2* cDNA^9^. This clarification eliminates the possibility that there has been a taxon-specific insertion/deletion event in *ASG2* involving sequence related to *ASG1*.

### Evolutionarily conserved elements of ASG2

Contigs matching *ASG2* were identified from all six target capture species. Within each species, the *ASG2* contigs shared > 97% nucleotide identity and thus we report only the longest contig assembled from each species (Table 2). Across species, no splice junctions were detected and despite the substantial 1,874–3,130 bp lengths of the contigs, no contig had a putative start codon. Instead, contigs were either entirely protein coding sequence or protein coding with a stop codon and 3′ UTR. All *ASG2* capture contigs are consistent with derivation from a large exon at the 3′ end of the gene or a large single exon gene.

**Figure 5:**
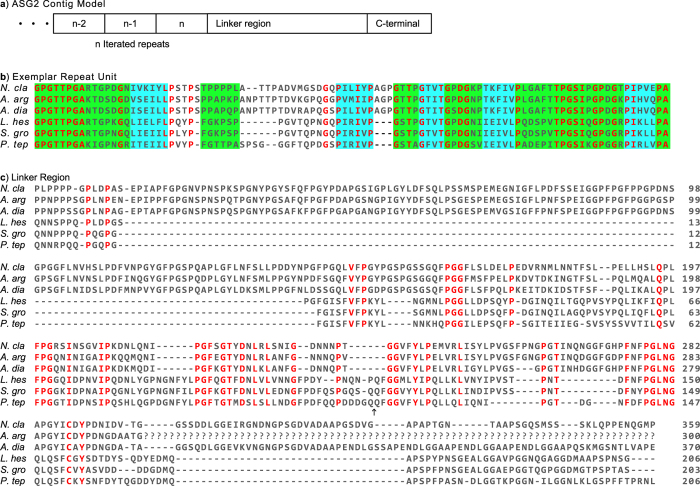
ASG2 from six species. (**a**) Schematic of ASG2 architecture inferred from our assembled sequences. Repetitive region contains an undetermined (n) number of repeat units followed by linker and carboxyl (C)-terminal regions. Ellipsis signifies unknown sequence. (**b**) Alignment of exemplar ASG2 repeat units indicating sequences predicted to form β**-**sheet (blue shading) or β-turn/random coil structure (green shading). (**c**) Alignment of linker regions. Amino acids depicted by single letter IUPAC abbreviations. Red indicates amino acids conserved across species and black arrow indicates location where additional sequence from *P. tep* whole genome assembly contig 63868 was added to our *P. tep* capture contig. The two sequences were 97% identical over 1874 bp. Abbreviations of species names and GenBank accession numbers are in Table 2.

**Figure 6:**
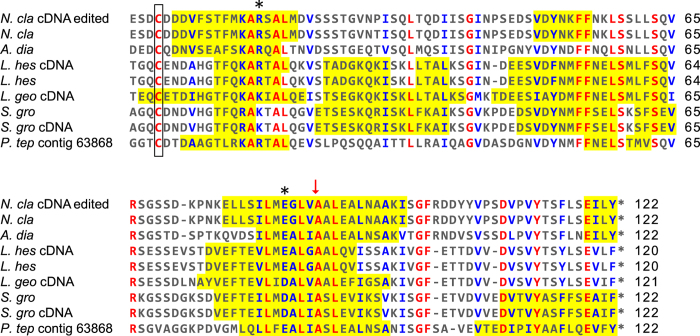
Alignment of ASG2 carboxyl-terminal regions. Predicted α-helical regions are highlighted in yellow, and red arrow shows location of removed sequence from *N. clavipes ASG2* cDNA (Fig. 4). Amino acids are depicted by single letter IUPAC abbreviations. Red indicates amino acids conserved across species and blue physiochemical conservation. Box encloses the conserved cysteines, and asterisks above columns indicate residues predicted to form salt bridges. Abbreviations of species names and GenBank accession numbers are in Table 2.

The inferred ASG2 proteins are readily aligned, revealing many conserved elements across species. The longest contigs encode proteins with three distinct regions: iterated repeats, linker, and carboxyl-terminal (Fig. 5a). First, present in all species is a repetitive region composed of tandem arrayed, repeat units of 89–99 amino acids, depending on species (Fig. 5b). Our contigs have at least three repeat units, but until a complete ASG2 gene or transcript is characterized, the total number of repeats within a repetitive region will remain unknown. Second, following the repetitive region is a “linker” (transitional) region of 200–300 amino acids (Fig. 5c). Lastly, there is a non-repetitive, carboxyl-terminal region of ~125 amino acids (Fig. 6).

The ASG2 repeat units are remarkably similar across species (Fig. 5b). In fact, 36 of 99 amino acids in the repeat unit alignment are conserved across species (Fig. 5b red residues). EMBOSS secondary structure prediction^23^,^24^ identified β-sheet, β-turn, and random coil forming sequences within the repeat units, but no α-helices. The various turn-coil-turn structures and β-sheets map identically across species on the amino acid alignment (Fig. 5b green and blue shading). Similar to fibrous spider silks, the periodic structure of the repeats may contribute to the elasticity and “suspension bridge” properties observed for viscous, aggregate silk droplets^5^,^8^. Furthermore, the abundant threonine residues (composing 9–15% of the repeat, depending on species) may provide O-glycosylation sites, which could explain the glycosylated proteins detected within the *N. clavipes* viscous droplets^9^.

Following the repetitive region is a non-repetitive linker region. The length of the linker region varies considerably across species, making them more difficult to align with each other than the exemplar repeats (Fig. 5). Despite the length differences, 40 residues are 100% conserved and many occur in adjacent positions. Juxtaposition of conserved sequence implies a potential structural or functional significance, such as separating the carboxyl-terminal region from the repetitive region.

### Conserved ASG2 carboxyl-terminal region

The ASG2 carboxyl-terminal region was captured from all species except *A. argentata* and *P. tepidariorum*. Both *A. argentata* and *P. tepidariorum* ASG2 contigs were truncated towards the end of the linker, before the start of the carboxyl-terminal region (Fig. 5c, black arrow and question marks). After removing the 423 bp insertion from the *N. clavipes ASG2* cDNA, its carboxyl-terminal region aligned well with the others (Fig. 6). Within species, the *ASG2* contigs from the biological replicates shared > 97% nucleotide identity, suggesting that unlike *ASG1*, *ASG2* is a single locus.

As was done with ASG1, we used BLAST with our target capture contigs to search the NCBI databases for ASG2 homologs. From the *P. tepidariorum* whole genome shotgun assembly, we located contig 63868, which possessed the ASG2 carboxyl-terminal region that was missing from our *P. tepidariorum* target capture contig (BLASTN e-value 0.0, 97% identity over 1,874 bp). BLASTN searches against the transcriptome shotgun assemblies for *L. hesperus* and *S. grossa* tissues, which included silk glands, also successfully yielded cDNAs that were near identical to our target capture contigs (*L. hesperus* e-value 0, 99% identity over 2,724 bp; *S. grossa* e-value 0, 98% identity over 1,984 bp). Additionally, TBLASTN searches found *ASG2* cDNA with a cross-species query against the *L. geometricus* transcriptome that was constructed from silk glands and other tissues (e-value 0, 85% identity over 945 amino acids). However, there were no matches to *ASG2* in the *S. mimosarum* genome (TBLASTX search, no matches with e-value < e-01). Although this absence may be due to incompleteness of the *S. mimosarum* genome assembly, absence of *ASG2* is also consistent with the lack of aggregate glands in *S. mimosarum*. Aggregate glands and ASG2 are likely to be evolutionary innovations that arose in a recent common ancestor of Araneoidea.

**Figure 7:**
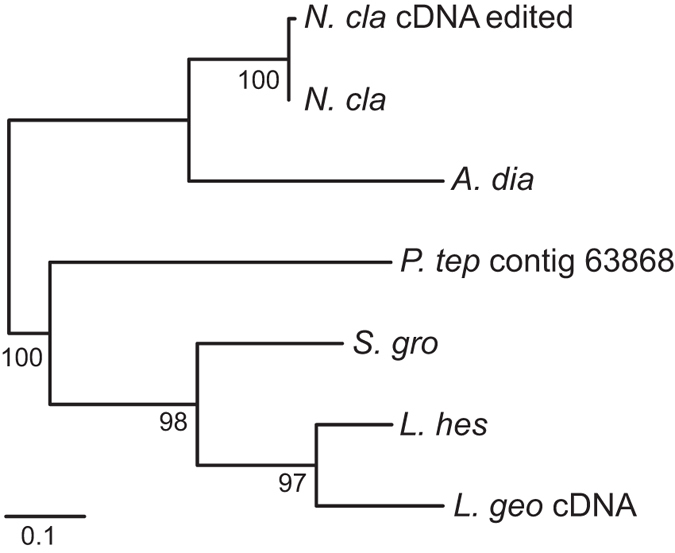
Maximum likelihood tree of ASG 2 carboxyl-terminal regions. When there was an identical match between a translated genomic contig and a cDNA transcript, only the genomic contig is shown in the tree (*L. hes* = *L. hes* cDNA, *S. gro* = *S. gro* cDNA). Abbreviations of species names and GenBank accession numbers are in Table 2. Bootstrap percentages > 50% are shown. Scale bar indicates 0.1 substitutions per site. Tree is mid-point rooted.

Phylogenetic analysis of the ASG2 carboxyl-terminal region resulted in one tree (Fig. 7). Relationships within Theridiidae mirrored phylogenetic relationships based on morphology^25^ and nuclear and mitochondrial sequences^26^. Specifically, 97% bootstrap support for a *Latrodectus* clade (*L. geometricus*, *L. hesperus*), 98% support for Latrodectines (*Latrodectus*, *S. grossa*), and 100% for Theridiidae (Latrodectines, *P. tepidariorum*). The strong clade support values and congruence of the gene tree with species-level relationships are further evidence that *ASG2* is a single locus gene.

There are numerous features of the carboxyl-terminal region that are conserved across species. Seventeen percent of the aligned amino acids (21 out of 123 positions) are invariant and another 19% (23/123) share physiochemical conservation (Fig. 6 red and blue, respectively), Given an estimated age of ~155 million years for Araneoidea^27^, purifying selection appears to have been maintaining these aspects of ASG2. The conserved residues are likely of structural and/or functional importance for prey capture. For example, the conserved cysteine (Fig. 6 orange box) could form a disulfide bridge with other ASG2 monomers, chemically linking them into polymers facilitating droplet extension to “suspension bridge” conformation^5^.

### ASG2 is a spidroin (spider fibroin)

Given the previously unrecognized carboxyl-terminal sequence similarities across species (Fig. 6), we investigated putative homologs for ASG2. We performed NCBI conserved domain searches with our ASG2 capture contigs and the edited *N. clavipes* ASG2 cDNA as queries, and found that the ASG2 carboxyl-terminal region matched the carboxyl-terminal regions of spidroins (Spidroin_MaSp pfam11260, e-values > 3.07e-3). DELTA-BLAST search with *N. clavipes* ASG2 resulted in a hit of e-value 2e-17 to major ampullate spidroin 1 (MaSp1) from *Argiope amoena* (AAP88232). Spidroins are a family of proteins that compose spider silks^28^. Spidroin gene family members tend to have coding regions > 10 kb that translate into > 200 kDa proteins^29^,^30^. Spidroin paralogs share a similar architecture that consists of an ~150 amino acid non-repetitive amino-terminal region, an extensive repetitive region, and an ~100 amino acid non-repetitive carboxyl-terminal region^30^. The amino acid sequence attributes of spidroin repetitive regions are associated with protein structures that underlie the impressive mechanical properties of spider silk fibers^31^.

The conserved domain and DELTA-BLAST searches with our ASG2 sequences resulted in hits to MaSp1 (see above). However, ASG2 repeat units (Fig. 5b) lack the distinguishing characteristics of MaSp1 paralogs (poly-alanine, glycine-alanine couplets, and glycine-glycine-X triplets)^32^. Instead, ASG2 is a newly discovered member of the spidroin gene family. We propose that ASG2 should be renamed aggregate spidroin 1 (AgSp1). As with other spidroins, given its extremely conserved architecture and sequence elements, the repetitive region of AgSp1 (ASG2; Fig. 5b) is also likely to be critical for the mechanical properties of viscous silk glues.

The AgSp1 (ASG2) repetitive region is followed by a linker region that varies in length across species (Fig. 5c), as has been observed in some spidroin family members^33^,^34^. Unlike the linker regions, spidroin carboxyl-terminal regions are conserved in length and structure^34^,^35^,^36^. The AgSp1 (ASG2) carboxyl-terminal region is no exception, with a series of conserved α-helices (Fig. 6, yellow highlights) that in other spidroins are described as forming a barrel-like structure^35^. In aciniform silk, interlocking spidroin dimers are stabilized by two salt bridges and a disulfide bond^36^. Consistent with this model, the AgSp1 (ASG2) monomers may be linked with salt bridges between the physiochemically conserved R/K and D/E residues (Fig. 6 asterisks) and disulfide bonds between the conserved cysteine residues (Fig. 6 orange box)^34^,^35^,^36^.

The recognition that ASG2 is a spidroin (AgSp1) integrates disparate observations of silk dope chemistry, aggregate silk gland morphology, and viscous glue droplet properties. For example, chemical composition of the viscous droplets resembles unprocessed silk dope of non-aggregate silk glands in terms of salts and up to 50% water content^37^. In the major ampullate silk glands, which produce dry, fibrous silk, salt and water molecules are removed during processing. The removal occurs as silk dope passes from the glandular lumen to the spigot via long and narrow ducts^38^. However, aggregate silk glands have distinctively short ducts connected to spigots with wide openings^39^. This means that silk dope could be secreted with minimal processing. The hypothesis that viscous droplets are composed of spidroin-based dope is supported by observations of droplet extension upon draw, crystallization upon dehydration, and a fibrous core to the viscous droplet^2^,^5^,^6^.

### Summary

By sequencing target capture genomic libraries, we report sequences for *ASG1* and *ASG2* from six spider species. We describe the gene structure of *ASG1* and recovered multiple gene variants in each individual, indicating that *ASG1* is multi-locus. Comparative analyses with homologs from the literature identified our ASG1 sequences as members of the peritrophic matrix intestinal mucin family. In insects, these mucins bind chitin in an extracellular matrix^12^. Furthermore, in the Western black widow, ASG1 is more highly expressed in non-silk gland tissue than in silk glands^11^. Thus, ASG1 is not a silk glue, but may have a role in providing structural reinforcement within silk glands, as well as other spider tissues.

Based on our target capture genomic contigs, we conclude that the previously reported *N. clavipes ASG2* cDNA is a chimeric sequence, and that the region with identity to *ASG1* appears to be a cloning artifact (Fig. 4b orange sequence). Because the artifactual sequence was inserted in the carboxyl-terminal region, the affinity of ASG2 to the spidroin gene family has been obscured until now. AgSp1 (aggregate spidroin 1) is a more appropriate name for ASG2, as ASG2 possesses the spidroin domain architecture of a repetitive region with tandem-arrayed repeat units, followed by a labile linker that leads to the conserved carboxyl-terminal region. Thus, aggregate silk glands produce viscous glue with a spidroin component. Realization that there is an aggregate spidroin deepens the mechanistic understanding of viscous glue droplet properties, such as formation of fibrous cores and viscoelastic-solid behaviour. The comparison of spidroin glue sequences from cob-web weavers and orb-web weavers revealed extensive evolutionary conservation of amino acid sequences and motifs. These biochemical features are relevant to research on bioinspired glues. Moreover, the discovery of a spidroin family member that is specialized to be a glue inspires future studies on the evolutionary reshaping of silk glands for production of dry fibers and wet adhesives.

## Methods

### Target Capture Probes

cDNA sequences for all annotated aggregate spider glues from *N. clavipes* (GenBank accessions EU780015, EU780014) and *L. hesperus* (JX262189, GBCS01010755, GBCS01018793, GBCS01005615, GBCS01005654) were collected from NCBI in June 2014. The nucleotide sequences were aligned and identical sequences were consolidated with Sequencher 4.2 (Gene Codes, Ann Arbor MI). The resulting set of *ASG1* and *ASG2* reference sequences were used to design 120-mer oligonucleotide target capture probes with 5X tiling by Agilent Inc. (Santa Clara, CA).

### Genomic DNA extraction

We obtained live *N. clavipes* (Nephilidae) from Gainesville, Florida; *Argiope argentata* (Araneidae) from Encinitas, California; *Araneus diadematus* (Araneidae) from Berkeley, California*; L. hesperus* (Theridiidae) from Riverside, California; *Steatoda grossa* (Theridiidae) from Yarnell, Arizona; and *Parasteatoda tepidariorum* (Theridiidae) from Lexington, Virginia. We sampled two individuals per species. Each spider was euthanized and its cephalothorax was placed in a microfuge tube, flash frozen in liquid nitrogen, and stored at −80 °C. Genomic DNA was extracted from each cephalothorax with the DNeasy Blood and Tissue kit (Qiagen, Valencia, CA) and treated with RNase A. DNA concentration was determined with a Qubit fluorometer (Life Technologies, Carlsbad, CA).

### Capture Library Construction

For each species, two target capture genomic libraries were constructed, for a total of twelve libraries. Each library represented the genome of an individual spider. The biological replicates were done to facilitate distinguishing allelic variation from multi-copy genes. Prior to library construction, 5 μg of DNA was sheared into ~500 bp fragments with an S220 focused-ultrasonicator (Covaris, Woburn, MA). The fragmented DNA was treated with PreCR Repair Mix (New England Biolabs, Ipswich, MA) and then purified with Agencourt AMPure XP beads (Beckman Coulter, Brea, CA). Library construction and target selection were performed with the SureSelect^XT^ Reagent Kit (Agilent), using the target capture probes described above. The libraries were sequenced bidirectionally (2 × 300bp) in multiplexed sets of four on a MiSeq instrument (Illumina, San Diego, CA) at the University of California, Riverside Genomics Core Facility.

### Contig Assembly

Prior to assembly, sequencing reads were filtered to minimize incorporation of low quality bases into the results. Using FASTX-toolkit (http://hannonlab.cshl.edu/fastx_toolkit/), each of the 24 fastq files (12 libraries, 2 directions each) was filtered separately. First, library construction adapters and indexing barcodes were removed from each read, and reads containing any ambiguous bases (basecalls other than A, C, G, T) were entirely discarded. For each fastq file, quality scores and base composition at each position were plotted. Plots were made with FastQC (http://www.bioinformatics.babraham.ac.uk/projects/fastqc/) implemented on the Galaxy webserver, usegalaxy.org^40^,^41^,^42^. Based on these plots, the first 6–10 bases were removed from the 5′ end of each read with FASTX-toolkit. These initial bases had lower quality and skewed base composition, attributed to artifacts from the library construction steps (e.g., A-tailing). Scanning from 5′ to 3′, reads were also trimmed from the earliest position with a first-quartile quality score below Q25. Trimmed reads with > 15% of their bases below quality score Q25 were removed (FASTX-toolkit). The filtered reads were sorted to retain only paired-end reads (Prinseq)^43^. Paired-end reads were interleaved into a single file before contig assembly, for a total of 12 quality-filtered fastq files (rackJ)^44^. The Trinity assembler was used to construct contigs from the quality-filtered fastq files^45^. Each library was assembled separately, using default parameters and paired-end reads.

### Sequence Analyses

Contigs representing homologs to *ASG1* or *ASG2* were identified by nucleotide similarity to the reference sequences (described above) using Geneious v. 6.18 (http://www.geneious.com)^46^. Only contigs with representatives from both individuals of a species were analyzed. Additional relevant contigs were also identified with BLASTX^47^ against the translations of the reference sequences. Putative contigs were then confirmed through BLASTX searches against the NCBI nr database.

Contigs representing homologs to *ASG1* or *ASG2* were translated *in silico*. Potential exon splice sites were annotated with the Splice Site Prediction tool at the Berkeley *Drosophila* Genome Project website using the “*Drosophila*” standard settings (http://www.fruitfly.org/seq_tools/splice.html)^48^. Amino acid compositions were calculated in Geneious and peptide secondary structures were predicted with the EMBOSS Geneious plugin^23^,^24^. TBLASTN searches against whole genome (*P. tepidariorum* and *Stegodyphus mimosarum*) and transcriptome (*L. hesperus*, *Latrodectus geometricus* and *S. grossa*) shotgun assembly databases on NCBI were conducted to identify additional relevant sequences for *ASG1* (Table 1) and for *ASG2* (Table 2). CDD (conserved domain database)^49^, DELTA-BLAST (domain enhanced lookup time accelerated BLAST)^16^, and CDART (conserved domain architecture retrieval tool)^20^ implemented on the NCBI website were used to annotate putative protein function. Sequences were translated and carboxyl-terminal regions aligned in Geneious using the Clustal W algorithm^50^, then refined by eye. Amino acid model testing and maximum likelihood gene tree construction with 10,000 bootstrap replicates were done with RAxML v8.2.X^51^. Amino acid substitution models FLU and JTT were used for ASG1 and ASG2 tree constructions, respectively. Resulting trees were mid-point rooted and visualized with FigTree v1.4.2 (http://tree.bio.ed.ac.uk/software/figtree/).

## Additional Information

**How to cite this article**: Collin, M. A. *et al.* Evidence from Multiple Species that Spider Silk Glue Component ASG2 is a Spidroin. *Sci. Rep.* **6**, 21589; doi: 10.1038/srep21589 (2016).

## Figures and Tables

**Table 1:**
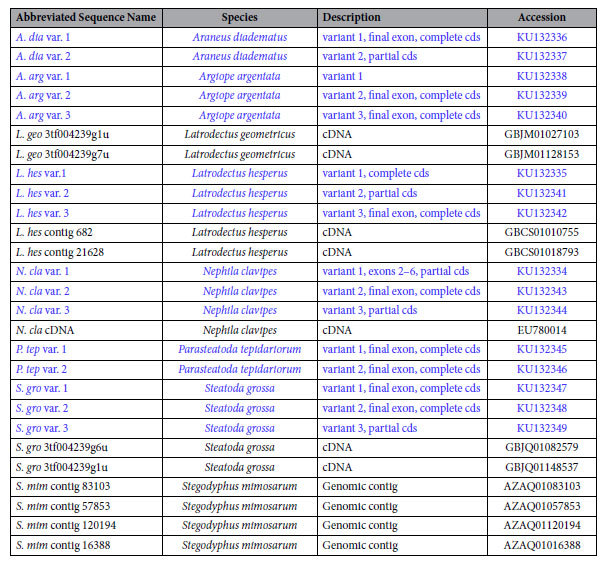
Description of *ASG1* sequences. Entries in blue are from this study.

**Table 2:**
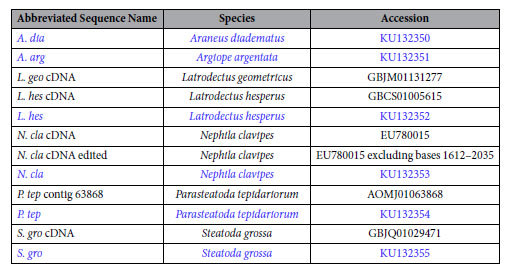
Description of *AgSp1*/*ASG2* sequences. Entries in blue are from this study.
